# Research on the Factors Influencing the Measurement Errors of the Discrete Rogowski Coil †

**DOI:** 10.3390/s18030847

**Published:** 2018-03-13

**Authors:** Mengyuan Xu, Jing Yan, Yingsan Geng, Kun Zhang, Chao Sun

**Affiliations:** State Key Laboratory of Electrical Insulation and Power Equipment, Xi’an Jiaotong University, Xi’an 710049, China; xumengyuan@stu.xjtu.edu.cn (M.X.); ysgeng@mail.xjtu.edu.cn (Y.G.); zk452332677@stu.xjtu.edu.cn (K.Z.); a932032472@stu.xjtu.edu.cn (C.S.)

**Keywords:** discrete Rogowski coil, mutual inductance, magnetic vector potential, interference error, eccentricity error

## Abstract

An innovative array of magnetic coils (the discrete Rogowski coil—RC) with the advantages of flexible structure, miniaturization and mass producibility is investigated. First, the mutual inductance between the discrete RC and circular and rectangular conductors are calculated using the magnetic vector potential (MVP) method. The results are found to be consistent with those calculated using the finite element method, but the MVP method is simpler and more practical. Then, the influence of conductor section parameters, inclination, and eccentricity on the accuracy of the discrete RC is calculated to provide a reference. Studying the influence of an external current on the discrete RC’s interference error reveals optimal values for length, winding density, and position arrangement of the solenoids. It has also found that eccentricity and interference errors decreasing with increasing number of solenoids. Finally, a discrete RC prototype is devised and manufactured. The experimental results show consistent output characteristics, with the calculated sensitivity and mutual inductance of the discrete RC being very close to the experimental results. The influence of an external conductor on the measurement of the discrete RC is analyzed experimentally, and the results show that interference from an external current decreases with increasing distance between the external and measured conductors.

## 1. Introduction

Current sensing technology is regarded as the foundation of intelligent electrical apparatuses and smart grids. Traditional electromagnetic current transformers exhibit deficiencies in many aspects, e.g., magnetic saturation, frequency response, and amplitude measurement range. The Rogowski coil (RC) has been very well known in laboratories for decades [1,2], especially for the measurement of high-frequency current pulses. The output voltage at the open terminals of the RC is proportional to the derivative of the current with respect to time. RC transducers have many advantages compared to electromagnetic current transformers: They can endure large overloads without damage, can measure currents over an extensive range without saturation, offer flexibility and light weight, and have low cost, wide bandwidth, and excellent transient response. The RC can be applied to measure currents in power systems, short-circuit testing systems, electromagnetic launchers, slip-ring inductance motors, and lightning test facilities.

Extensive analytical electromagnetic modeling of the traditional RC and of the effects of non-idealities can be found in a few recent papers [3,4,5,6,7] and innovative RC designs have been proposed recently using printed circuit board technology [8]. Unfortunately, miniaturizing the inductance coil sensors is rather difficult because their sensitivity depends on the sensor area (or the length of the core). Nevertheless, micro coil sensors with dimensions <1 mm that have been prepared through the use of thin film techniques are reported. Another relative low-cost solution is the discrete RC [9].

**Figure 1:**
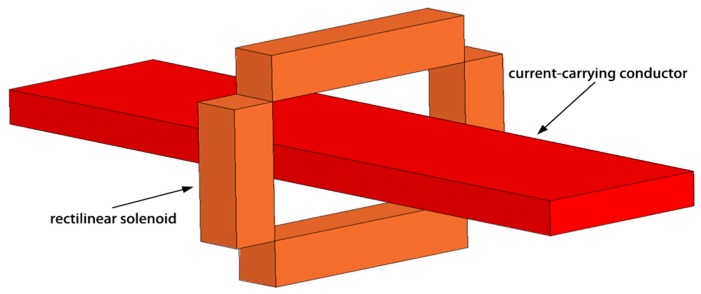
Model of a discrete RC made of four rectilinear solenoids.

In many industrial applications (such as current measurement in moulded case circuit breakers (MCCBs)), RCs of small dimensions are needed for mass production. Due to the limitation of its structure and processing technology, the traditional loop coil cannot meet the requirements of mass production, low cost and miniaturization. Because fabricating a rectilinear solenoid is much easier and less expensive than making a toroidal one (as the traditional RC), it is of interest to investigate the performance of what can be defined as a “discrete” RC. This RC is composed of some rectilinear solenoids connected in series [10], as shown in Figure 1.

In this paper, an innovative array of magnetic coils (the discrete RC) with the advantages of low cost, miniaturization, and mass producibility is investigated for applications to MCCB with rated current 400 A and frequency 50 Hz in power system. First, the mutual inductance between a discrete RC and circular and rectangular conductors are calculated with the help of the magnetic vector potential (MVP) method, and the correctness of this method is then verified by using the finite element method. Then, the influences of section parameters, inclination, eccentricity, external currents, and number of solenoids on the measurement accuracy of discrete RC is analyzed by using MATLAB software, which provides a reference for engineering applications. Finally, tests on the discrete RC are conducted, the results of which show that the current transducer based on the discrete RC has a stable sensitivity and that the theoretically calculated values by using the MVP method match well with the experimentally measured values. Through tests, the performance of the discrete RC is verified and the influence on its accuracy caused by an external conductor is investigated and compared with the analytical results.

## 2. MVP Method for Mutual Inductance Computation

The mutual inductance between a conventional RC and a conductor can be calculated by using Ampere’s law of total current for its closed structure. However, Ampere’s law cannot be used to calculate the mutual inductance of a discrete RC as it is an unclosed structure. Here, the mutual inductance is deduced by using the MVP method [11,12]. 

In a constant magnetic field, the magnetic field strength **B** is divergence free, and the magnetic vector potential **A** can be introduced to facilitate the calculation of the magnetic field **B**. The relation between **B** and **A** is:

(1) B=∇×A.

The magnetic vector potential **A** of the conductor in space is:

(2) $$A=A_{x}e_{x}+A_{y}e_{y}+A_{z}e_{z}=\frac{\mu }{4\pi }\int_{l}\frac{Idl}{r},$$

where *μ* is the permeability, **I** is the current flowing through the conductor, and *r* is the vertical distance from one point in space to the conductor. 

**Figure 2:**
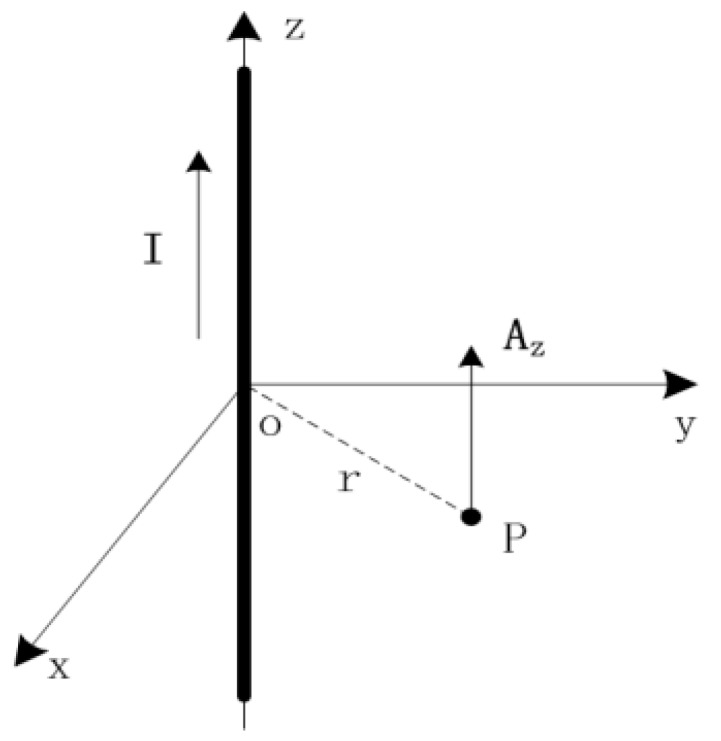
Magnetic vector potential **A** at one point in space.

When the length of the conductor is a factor of 5 greater than the diameter of the coil, the conductor can be considered infinite. From (2), we know that the magnetic vector potential **A** is parallel to **I***dl*. As shown in Figure 2, if the current flowing through the conductor is along the *z* axis, the magnetic vector potential **A** at one point in space is simply *A_z_*.

If the conductor has a circular cross section with a radius *R*, the magnetic vector potential *A_z_* of point (*x*, *y*) is:

(3) $$A_{z}=\frac{\mu I}{2\pi }\ln\frac{\sqrt{x^{2}+y^{2}}}{R},$$

where *μ* is the permeability and *I* is the current flowing through the conductor. 

If the conductor has a rectangular cross section with a width 2*a* and a height 2*b*, the magnetic vector potential *A_z_* at point (*x*, *y*) is [13]:

(4) $$\begin{array}{ll} A_{z}=\frac{\mu I}{16\pi ab} & \cdot \{\left(a-x\right)\cdot \left(b-y\right)\cdot \ln\left[{\left(a-x\right)}^{2}+{\left(b-y\right)}^{2}\right]\\ & +\left(a+x\right)\cdot \left(b-y\right)\cdot \ln\left[{\left(a+x\right)}^{2}+{\left(b-y\right)}^{2}\right]\\ & +\left(a-x\right)\cdot \left(b+y\right)\cdot \ln\left[{\left(a-x\right)}^{2}+{\left(b+y\right)}^{2}\right]\\ & +\left(a+x\right)\cdot \left(b+y\right)\cdot \ln\left[{\left(a+x\right)}^{2}+{\left(b+y\right)}^{2}\right]\\ & +{\left(a-x\right)}^{2}\cdot \left[\arctan\left(\frac{b-y}{a-x}\right)\right]+\arctan\left(\frac{b+y}{a-x}\right)\\ & +{\left(a+x\right)}^{2}\cdot \left[\arctan\left(\frac{b-y}{a+x}\right)\right]+\arctan\left(\frac{b+y}{a+x}\right)\\ & +{\left(b-y\right)}^{2}\cdot \left[\arctan\left(\frac{a-x}{b-y}\right)\right]+\arctan\left(\frac{a+x}{b-y}\right)\\ & +{\left(b+y\right)}^{2}\cdot \left[\arctan\left(\frac{a-x}{b+y}\right)\right]+\arctan\left(\frac{a+x}{b+y}\right)\}. \end{array}$$

The discrete RC is composed of *N* rectilinear solenoids connected in series. After one calculates the mutual inductance between the conductor and the rectilinear solenoid, *M_i_*, the mutual inductance of the conductor and the whole coil, *M*, can be calculated by using the following equation:

(5) $$M=\sum_{i=1}^{N}M_{i},$$

where *N* is the number of solenoids and *M_i_* is the mutual inductance between the conductor and the rectilinear solenoid. 

**Figure 3:**
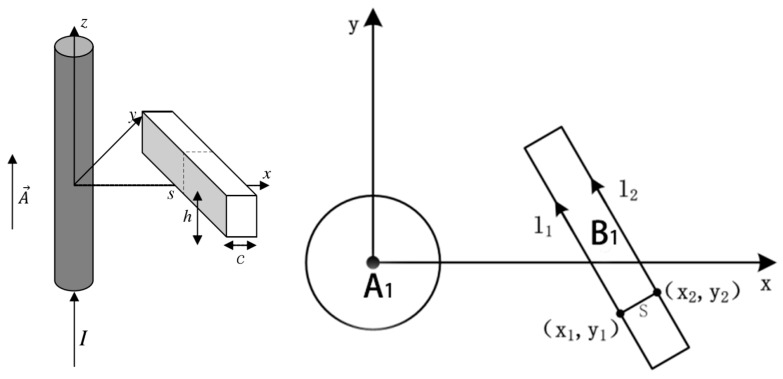
Model of the conductor and rectilinear solenoid.

In Figure 3, **A**_1_ represents the measured conductor, and the current is a constant current along the *z* axis. **B**_1_ represents a rectilinear solenoid with a rectangular cross section. **l_1_** and **l_2_** are the integral path of (*x*_1_, *y*_1_) and (*x*_2_, *y*_2_) along the solenoid. According to Stokes’ theorem, the magnetic flux Φ through surface *S* can be calculated by using the following equation:

(6) $$\Phi =\int_{S}B\cdot dS=\int_{S}\nabla \times A\cdot dS=\oint_{l_{c}}A\cdot dl,$$

where *S* and *l_c_* are the cross section of the rectilinear solenoid and the closed path along the section, respectively. 

Because the solenoid winding is not ideal, the cross section of the coil winding should be considered as a finite value in the actual calculation of flux through the rectangular solenoid. Thus, the flux linkage of a solenoid cannot be simply calculated by the sum of *N* coil magnetic fluxes. If one assumes that the solenoid winding is uniform, then one can take an infinitesimal *dl* on the rectilinear solenoid, and each turn on the *dl* can be considered infinitely thin. 

The total magnetic chain Ψ on the rectilinear solenoid of is:

(7) $$\begin{array}{ll} d\Psi & =dN\cdot \int_{S}B\cdot dS=dN\cdot \oint_{l_{c}}A\cdot dl\\ & =\frac{N}{L}dl\cdot \left[A_{z}\left(x_{2},y_{2}\right)-A_{z}\left(x_{1},y_{1}\right)\right]\cdot h \end{array},$$

where *N* is the number of solenoids, *L* is the length of each solenoid, and *h* is the height of each solenoid in the *z* axis direction. 

The total flux linkage of the whole solenoid is:

(8) $$\Psi =\frac{Nh}{L}\left[\int_{l_{2}}A_{z}\left(x_{2},y_{2}\right)\cdot dl-\int_{l_{1}}A_{z}\left(x_{1},y_{1}\right)\cdot dl\right].$$

The mutual inductance between the rectilinear solenoid and the conductor is:

(9) $$\begin{array}{ll} M_{i} & =\frac{\Psi }{I}=\frac{Nh}{IL}\int_{l_{2}}A_{z}\left(x_{2},y_{2}\right)\cdot dl\\ & -\frac{Nh}{IL}\int_{l_{1}}A_{z}\left(x_{1},y_{1}\right)\cdot dl. \end{array}$$

The integrals in Equation (9) can be calculated numerically. The mutual inductance of the total solenoid is then the sum of the mutual inductance of each individual solenoid. 

## 3. Comparison between the MVP and Finite Element Methods

To verify the accuracy of the MVP method for calculating mutual inductance, models of a conductor and a discrete RC using ANSYS Maxwell software were built to calculate the coefficient of mutual inductance [14].

### 3.1. Mutual Inductance between the Discrete RC and a Circular Conductor

**Figure 4:**
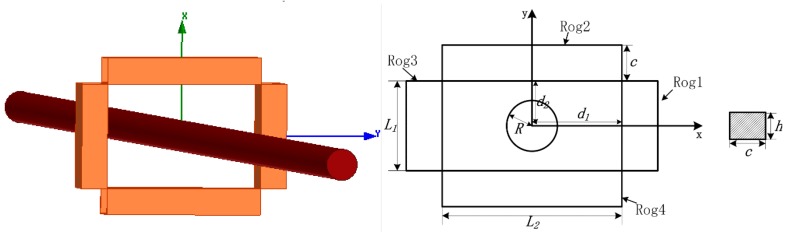
Model of a circular conductor and a discrete RC, with *R* = 3 mm, *c* = 4 mm, *h* = 3 mm, *L*_1_ = 10 mm, *L*_2_ = 20 mm, *d*_1_ = 10 mm, *d*_2_ = 5 mm, *ρ* (winding density) = 50 turns/mm.

The model of a circular conductor and four discrete solenoid coils is shown in Figure 4. The primary current *i*_1_ = 400√2 × sin(100π*t*). The frequency is 50 Hz. The subsequent simulations are all calculated with this primary current. Table 1 and Table 2 list the mutual inductance calculated using the MVP method and the finite element method. The total mutual inductance calculated using the MVP method is 6.5370 × 10^−7^ H, while the mutual inductance calculated using the finite element method is 6.4603 × 10^−7^ H. The relative error is only 1.19%. 

### 3.2. Mutual Inductance between the Discrete RC and a Rectangular Conductor

**Figure 5:**
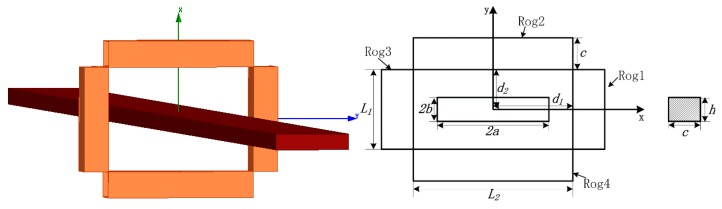
Model of a rectangular conductor and a discrete RC, with 2*a* = 15 mm, 2*b* = 3 mm, *c* = 4 mm, *h* = 3 mm, *L*_1_ = 10 mm, *L*_2_ = 20 mm, *d*_1_ = 10 mm, *d*_2_ = 5 mm, *ρ* = 50 turns/mm.

The model of a rectangular conductor and four discrete solenoids coil is shown in Figure 5. Table 3 and Table 4 list the coefficient of mutual inductance calculated using the MVP method and the finite element method. The total mutual inductance calculated using the MVP method is 6.4655 × 10^−7^ H, while the mutual inductance calculated using the finite element method is 6.3867 × 10^−7^ H. The relative error is only 1.23%. 

A comparison of the two methods demonstrates that the MVP method can accurately calculate the mutual inductance between a discrete RC and a conductor. Compared with the finite element method, the MVP method is simpler and more practical, making it suitable for the design calculation of a discrete RC. 

## 4. Influence of Parameters and Positions of Conductor on Mutual Inductance

According to Equation (9), the section parameters of the conductor and the relative position between the conductor and the coil will have some impact on the mutual inductance [15]. 

### 4.1. Influence of the Section Parameters of the Conductor on Mutual Inductance

**Figure 6:**
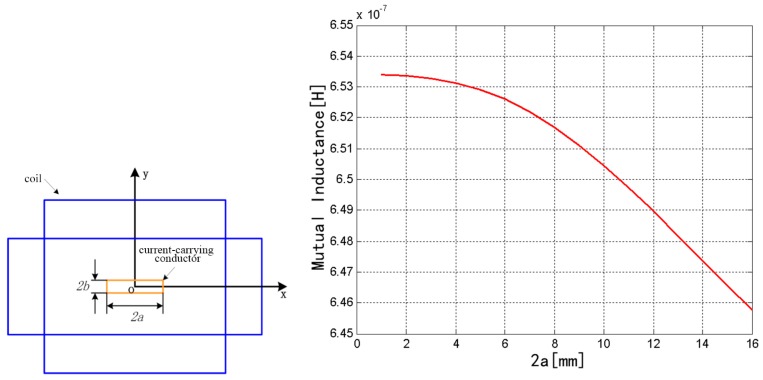
Influence of rectangular conductor parameters on mutual inductance.

Here, only the effect of changes in the parameters of the rectangular cross section on the mutual inductance was discussed. In the model established in Maxwell, 2*b* was fixed to 3 mm and 2*a* was changed from 1 to 16 mm. The change of mutual inductance is shown in Figure 6. When 2*a* was changed from 1 to 16 mm, the mutual inductance decreased from 6.5339 × 10^−7^ to 6.4576 × 10^−7^ H. Therefore, the coefficient of mutual inductance exhibits a decreasing trend with the increase of the width of rectangular section 2*a* over a certain range.

### 4.2. Influence of the Inclination of the Conductor on Mutual Inductance

In the process of coil installation, there may be a relative inclination between the discrete RC and the conductor. According to the inclined direction of the conductor, one can simply divide the influence of the inclination into rotations about the *x*, *y*, and *z* axes.

#### 4.2.1. Conductor with the *x* Axis Taken as the Rotation Axis

**Figure 7:**
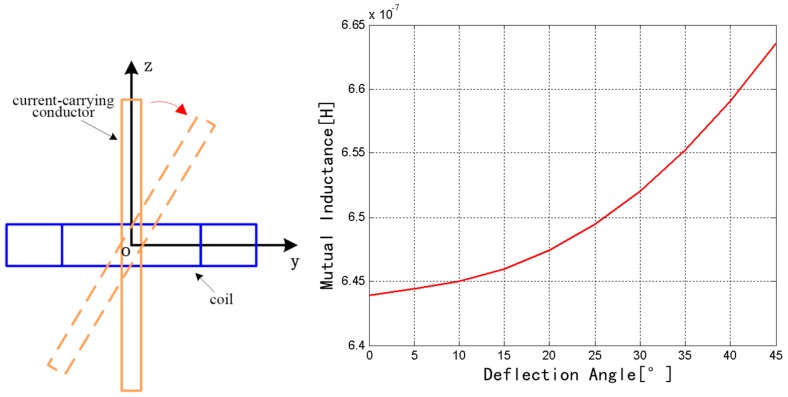
Change of mutual inductance with the *x* axis as the rotation axis.

The discrete RC was fixed, and the rectangular conductor rotates around the *x* axis from 0° to 45°, as shown in Figure 7. The mutual inductance increases from 6.4393 × 10^−7^ to 6.6354 × 10^−7^ H. Therefore, the mutual inductance exhibits an increasing trend with the increase of the rotation angle. 

#### 4.2.2. Conductor with the *y* Axis Taken as the Rotation Axis

**Figure 8:**
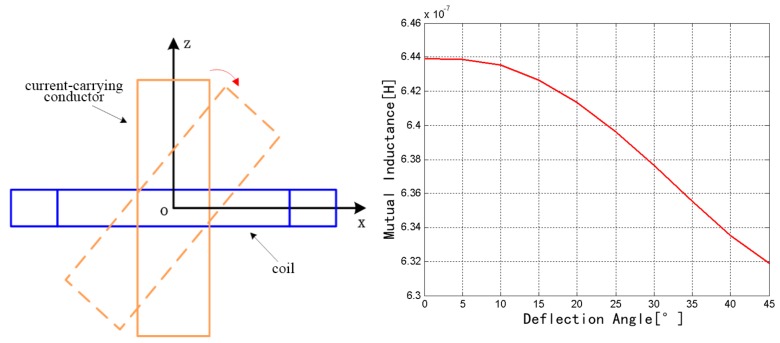
Change of mutual inductance with the *y* axis as the rotation axis.

The coil was fixed, and the rectangular conductor rotates around the *y* axis from 0° to 45°, as shown in Figure 8. The mutual inductance decreases from 6.4393 × 10^−7^ to 6.3192 × 10^−7^ H. Therefore, the mutual inductance exhibits a decreasing trend with the increase of rotation angle. 

#### 4.2.3. Conductor with the *z* Axis Taken as the Rotation Axis

**Figure 9:**
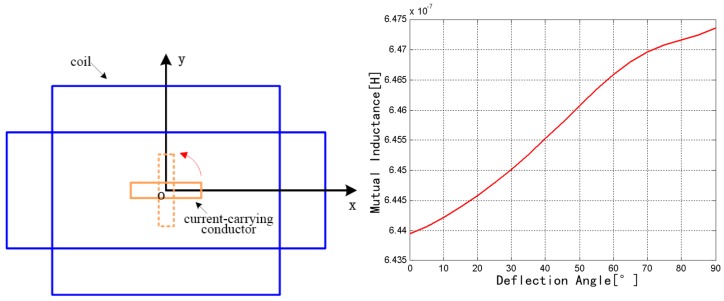
Change of mutual inductance with the *z* axis as the rotation axis.

The coil was fixed, and the rectangular conductor rotates around the current direction from 0° to 90°, as shown in Figure 9. The mutual inductance increases from 6.4393 × 10^−7^ to 6.4735 × 10^−7^ H. Therefore, the mutual inductance exhibits an increasing trend with the increase of rotation angle. 

### 4.3. Influence of the Eccentricity of the Conductor on Mutual Inductance

**Figure 10:**
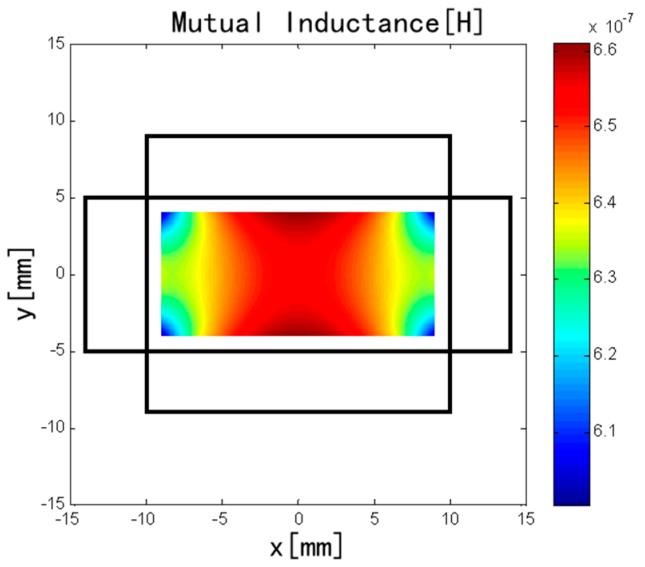
Influence of eccentricity of the conductor on mutual inductance.

Eccentricity of the conductor can also easily occur during coil installation. Because the discrete RC does not meet the conditions of Ampere’s law, the eccentricity of the conductor will have some impact on mutual inductance [16]. For convenience, the current carrying conductor is simplified as one point in the rectangular area, and the eccentricity error was studied by MVP method. The distribution of the mutual inductance is shown in Figure 10 when the conductor changes in rectangular area from −9 to 9 mm in the *x* range and from −4 to 4 mm in the *y* range. The mutual inductance is greater when the conductor is closer to the central region of a long solenoid, and the mutual inductance is smaller when the conductor is closer to the central region of a short solenoid.

## 5. Interference Error Caused by an External Current

### 5.1. Calculation of the Interference Error

When there is an external current outside the discrete RC, some errors will certainly occur in the measurement of the current encircled by the discrete RC. This is due to the error in the approximation of Ampere’s law from the incomplete closed geometry of the discrete RC. In the actual measurement process, the value of the external current and the relative position of the external conductor will lead to interference errors. In fact, in AC conditions, the output voltage of the discrete RC is given by:

(10) $$\dot{e}=j\omega M\dot{I}+j\omega M^{\prime }{\dot{I}}^{\prime },$$

where *ω* is the angular frequency of the AC, *M* is the mutual inductance between the discrete RC and the conductor, *M*′ is the mutual inductance between the RC and the external conductor, *İ* is the phasor of the current under measurement, and *İ*′ is the phasor of an external current flowing in a filamentary conductor parallel to the conductor. 

The sensitivity of the discrete RC is:

(11) S=jωM,

The value of the current to be measured is obtained as follows:

(12) $$\hat{\dot{I}}=\frac{\dot{e}}{S}=\dot{I}+\frac{M^{\prime }}{M}{\dot{I}}^{\prime }.$$

The relative error is given by:

(13) $$\varepsilon =\left|\frac{\hat{\dot{I}}-\dot{I}}{\dot{I}}\right|=\left|\frac{M^{\prime }}{M}\cdot \frac{{\dot{I}}^{\prime }}{\dot{I}}\right|.$$

When *İ*’= *İ*, the relative error is:

(14) $$\varepsilon =\left|\frac{M^{\prime }}{M}\right|\times 100\%.$$

**Figure 11:**
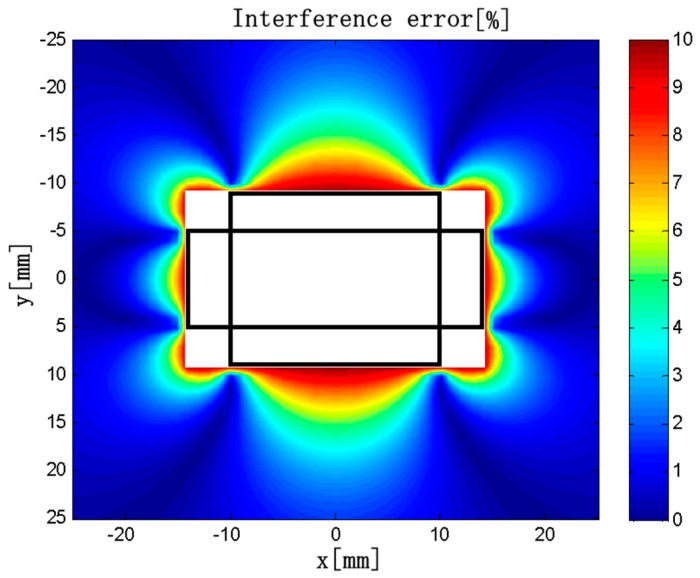
Interference error distribution.

As an example, a discrete RC composed of four solenoids as represented in Figure 5 is taken into account. The dimensions of the solenoids are *c* = 4 mm, *h* = 3 mm, *L*_1_ = 10 mm, *L*_2_ = 20 mm, *ρ* = 50 turns/mm, *d*_1_ = 10 mm, and *d*_2_ = 5 mm. The current carrying conductor is a rectangular conductor with a cross section *2a* × *2b* = 15 × 3 mm, which is placed in the center. The external conductor is a circular conductor with a diameter of 1 mm. The current of the two conductors are all 400√2 × sin(100π*t*). The frequency are all 50 Hz. The interference error distribution is shown in Figure 11. 

### 5.2. Influence of the Length of the Solenoids

To investigate the effects of the geometric parameters and arrangement position of the discrete RC on the interference error caused by the external magnetic field, we set the parameters and position of the long solenoids and analyzed the variation of the interference error when the length, winding density, and arrangement position of the short solenoids were changed.

**Figure 12:**
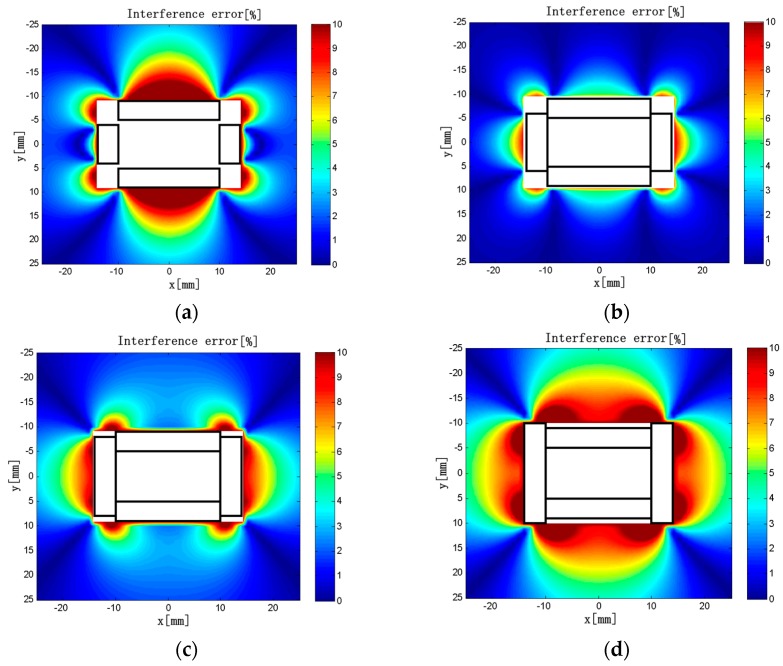
(**a**) *L*_1_ = 8 mm; (**b**) *L*_1_ = 12 mm; (**c**) *L*_1_ = 16 mm; (**d**) *L*_1_ = 20 mm. Interference error distributions (*L*_1_ = 8–20 mm).

Keeping *L*_2_ = 20 mm, *ρ*_1_ = *ρ*_2_ = 50 turns/mm, *d*_1_ = 10 mm, *d*_2_ = 5 mm, and *c* × *h* = 4 × 3 mm unchanged in the model, we obtained the interference error distributions is shown in Figure 12 when *L*_1_ = 8, 12, 16, and 20 mm.

As shown in Figure 12, the region with greater interference error tends to decrease first and then increase with the increase of *L*_1_. When *L*_1_ = 8 mm, the interference error is >10% in the vicinity of the solenoids. When *L*_1_ = 12 mm, the interference error in the vicinity of the long solenoids is ~4%, and the influence of the external current is acceptable here. The maximum error is 8.5%, which is the smallest in all cases. When *L*_1_ = 16 mm, the interference error is <4% in the vicinity of the long solenoids. In contrast to that of *L*_1_ = 12 mm, the region with large interference error is larger near the short solenoids, and the interference of the external current is more obvious. When *L*_1_ = 20 mm, the interference error is very large. The maximum interference errors are listed in Table 5 when *L*_1_ is varied in the range of 6 to 20 mm. 

Based on the above analysis and the maximum interference error distributions under different conditions, when *L*_1_ = 12 mm, the influence of the external current on measurement accuracy of the discrete RC is minimal. The main reason is that the overlapping or the gap between solenoids is the most suitable in all cases, which can minimize *M*′.

### 5.3. Influence of the Winding Density of the Solenoid

**Figure 13:**
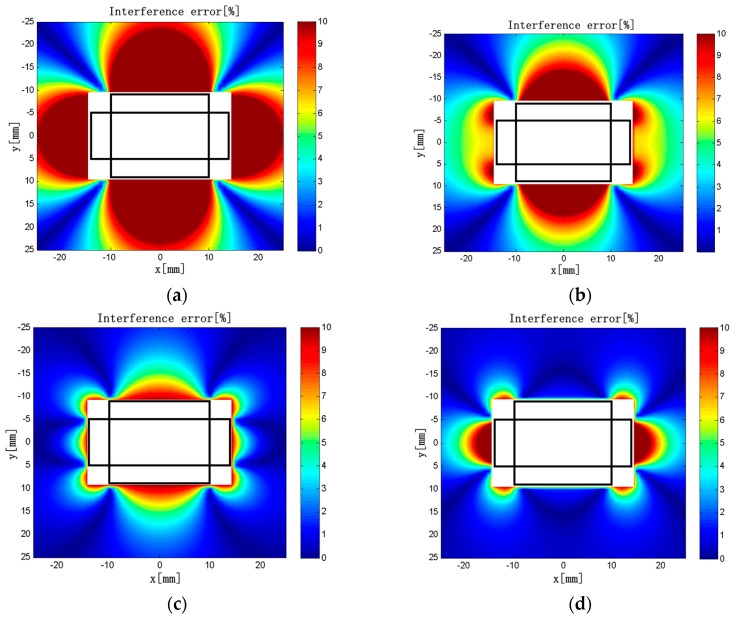
(**a**) *ρ*_1_ = 10 turns/mm; (**b**) *ρ*_1_ = 30 turns/mm; (**c**) *ρ*_1_ = 50 turns/mm; (**d**) *ρ*_1_ = 70 turns/mm. Interference error distributions (*ρ*_1_ = 10–70 turns/mm).

Keeping *L*_1_ = 10 mm, *L*_2_ = 20 mm, *ρ*_2_ = 50 turns/mm, *d*_1_ = 10 mm, *d*_2_ = 5 mm, and *c* × *h* = 4 × 3 mm unchanged in the model, we varied the winding density of the short solenoids in the range of 10–80 turns/mm. The interference error distributions are shown in Figure 13. 

The maximum interference errors are listed in Table 6. It can be seen that the interference error near the short solenoids tends to decrease first and then increase with the increase of *ρ*_1_ in a certain range. The interference error near the long solenoids tends to decrease with the increase of *ρ*_1_. When *ρ*_1_ = 50 turns/mm, the maximum interference error is only 9.3%, which is the smallest in all cases.

### 5.4. Influence of the Arrangement Position of the Solenoids

**Figure 14:**
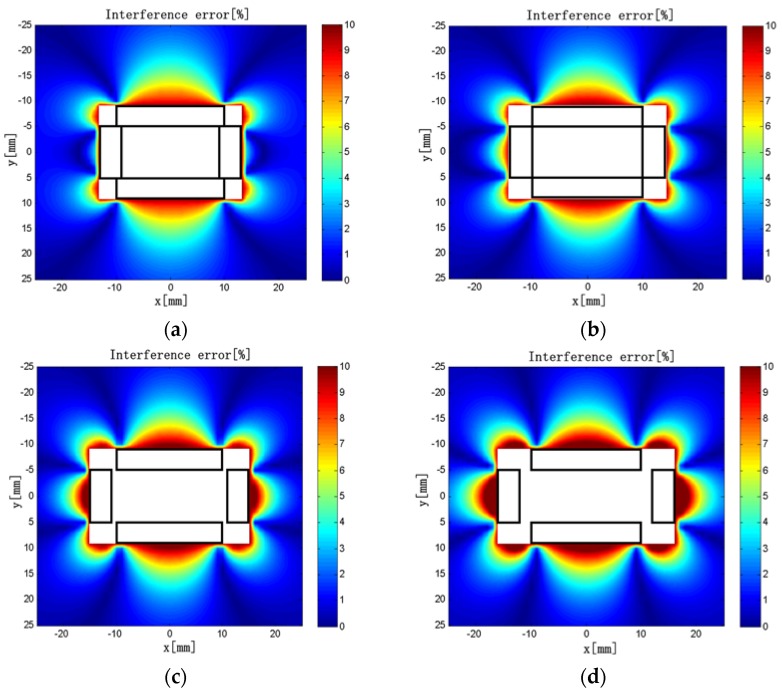
(**a**) *d*_1_ = 9 mm; (**b**) *d*_1_ = 10 mm; (**c**) *d*_1_ = 11 mm; (**d**) *d*_1_ = 12 mm. Interference error distributions (*d*_1_ = 9–12 mm).

Keeping *L*_1_ = 10 mm, *L*_2_ = 20 mm, *ρ*_1_ = *ρ*_2_ = 50 turns/mm, *d*_2_ = 5 mm, and *c* × *h* = 4 × 3 mm unchanged in the model, we varied the distance from the center to the short solenoids in the range of 8.5–12 mm. The interference error distributions are shown in Figure 14 when *d*_1_ = 9, 10, 11, and 12 mm. 

The maximum interference errors are listed in Table 7. 

It can be seen that the interference error near the solenoids tends to increase with the increase of *d*_1_ in a certain range. The maximum interference error decreases first and then increases with the increase of *d*_1_. When *d*_1_ = 10 mm, the maximum interference error is only 9.3%, which is the smallest in all cases. Therefore, when installing the discrete RC, the short solenoids should be secured to be close to the long solenoids to minimize the maximum interference error. 

## 6. Influence of the Number of Solenoids on Measurement Accuracy

**Figure 15:**
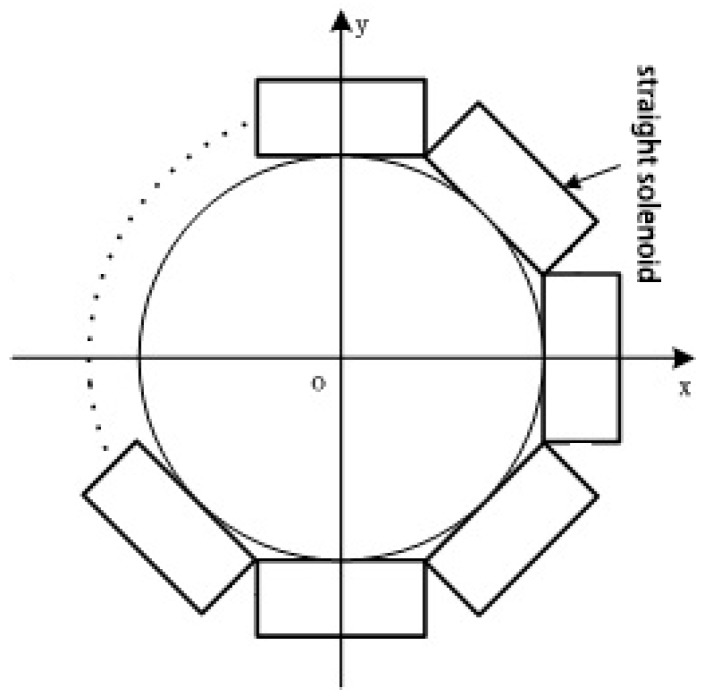
A discrete RC of *N* solenoids.

As is foreseeable, increasing the number of solenoids can make a discrete RC more similar to a traditional circular RC. As shown in Figure 15, in extreme cases, when *N*→∞, the discrete RC can be seen as a circular RC, Ampere’s law is completely applicable to it, and the eccentricity and interference errors of the discrete RC will tend to zero. In the following simulation, making *N* = 4, 6, 8 and 10, the straight solenoids with equal length are arranged around an inscribed circle with a diameter of 10 mm, respectively. *ρ* = 50 turns/mm. *c* × *h* = 4 × 3 mm.

### 6.1. Influence of the Number of Solenoids on Eccentricity Error

When the conductor is not in the center of the coil, an eccentricity error will be produced and this can be expressed by:

(15) $$\varepsilon =\frac{\left|M_{p}-M\right|}{M}\times 100\%,$$

where *M_p_* is the mutual inductance for the eccentric position and *M* is the mutual inductance for the center. 

**Figure 16:**
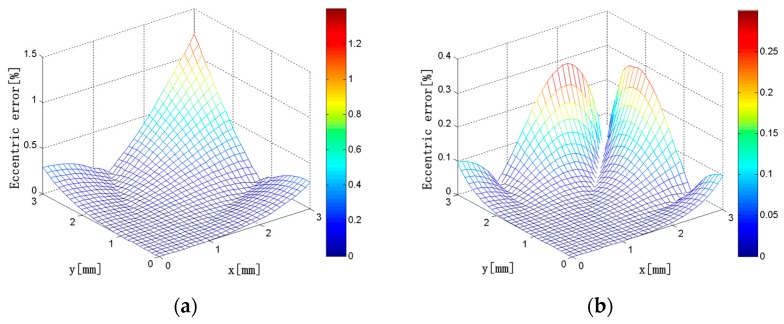

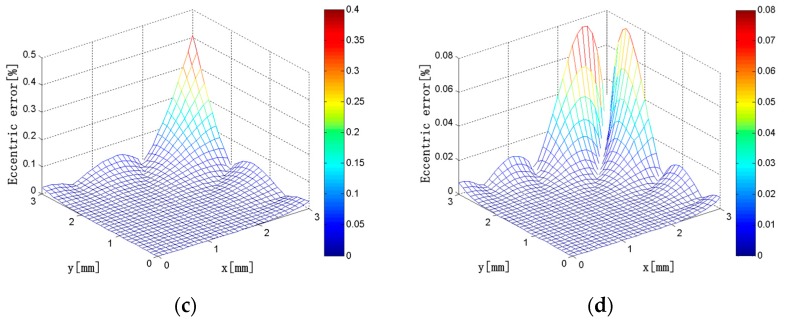
(**a**) *N* = 4; (**b**) *N* = 6; (**c**) *N* = 8; (**d**) *N* = 10. Eccentricity error distributions (*N* = 4, 6, 8, 10; *x* = 0–3 mm; *y* = 0*–*3 mm).

In this simulation, the current carrying conductor is a circular conductor with a diameter of 1 mm. The eccentricity error distributions of the discrete RC composed of *N* solenoids are shown in Figure 16 when the center of the conductor is changed in the square region where *x* = 0 to 3 mm and *y* = 0 to 3 mm in the first quadrant. It can be seen that, when *N* = 4, the maximum eccentricity error is 1.2%, and the eccentricity error is below 0.5% in most areas. As the number of the solenoids increases, the eccentricity error decreases gradually. When *N* = 10, the maximum eccentricity error is only 0.08%, and the eccentricity error is maintained below 0.02% in most areas. Therefore, with the increase of the number of solenoids, Ampere’s law is more and more applicable, and the eccentricity error of the discrete RC gets smaller and smaller. 

### 6.2. Influence of the Number of Solenoids on Interference Error

**Figure 17:**
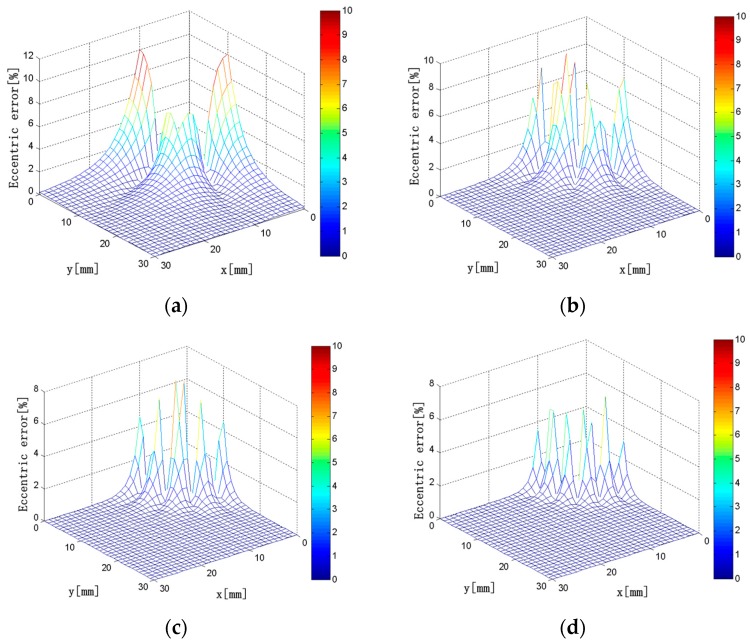
(**a**) *N* = 4; (**b**) *N* = 6; (**c**) *N* = 8; (**d**) *N* = 10. Interference error distributions (*N* = 4, 6, 8, 10; *x* = 0–30 mm; *y* = 0–30 mm).

In this simulation, the current carrying conductor and the external conductor are all circular conductors with a diameter of 1 mm. The interference error of the external current on the discrete RC can be calculated using Equation (14). When the external current is changed in the square region where *x* = 0 to 30 mm and *y* = 0 to 30 mm in the first quadrant, the interference error distributions of the discrete RC composed of *N* solenoids are shown in Figure 17. It can be seen that, when *N* = 4, the maximum interference error is 10%, and the interference error is below 6% in most areas. As the number of solenoids increase, the interference error decreases gradually. When *N* = 10, the maximum interference error is only 6%, and the interference error is basically zero with the exception of a few regions outside the discrete RC. Therefore, with the increase of the number of solenoids, Ampere’s law is more and more applicable, and the interference error of the discrete RC gets smaller and smaller. 

## 7. Experimental Research on the Discrete RC

### 7.1. Experiment Setup

**Figure 18:**
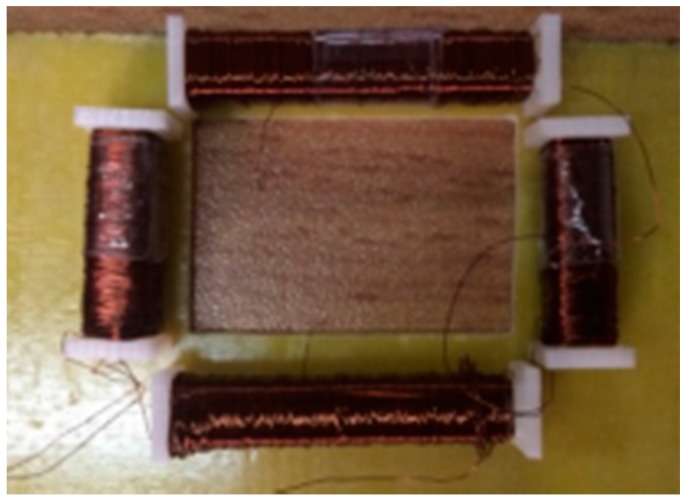
Discrete RC prototype.

The physical model of the discrete RC is shown in Figure 18. Size parameters are as follows: the short solenoid size is *L*_1_ × *c* × *h* = 12 × 5 × 4 mm^3^, the long solenoid size is *L*_2_ × *c* × *h* = 20 × 5 × 4 mm^3^, *d*_1_ = 10 mm, *d*_2_ = 6 mm, and the winding turns is 50 turns/mm. 

**Figure 19:**
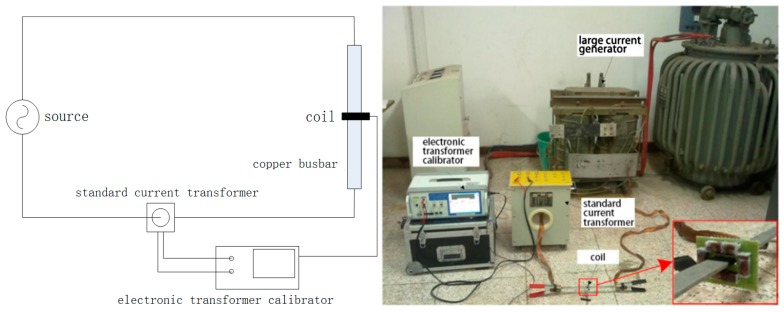
Test circuit for a discrete RC.

The test circuit of a discrete RC is shown in Figure 19; it includes a large current generator, a standard current transformer, a test coil, and an electronic transformer calibrator. The XL807 electronic transformer calibrator (Shenzhen Xinglong Technology Company, Shenzhen, China) contains a 24-bit analog-to-digital converter and can realize high accuracy within 0.05%. 

### 7.2. Performance Tests of the Discrete RC

The performance tests of the discrete RC include a sensitivity test, a conformance test, and a phase difference test. Sensitivity is an important index to measure the performance of the RC, and it is equal to the ratio of the output voltage to the primary current. The sensitivity of the discrete RC must be kept at a relatively stable value to accurately reflect the change of the primary current. In this experiment, the XL807 electronic transformer calibrator was used to measure the phase difference between the induced voltage of the discrete RC and the primary current.

#### 7.2.1. Circular Conductor Experiment

In this experiment, the diameter of the conductor is 5 mm, and the primary current is generated by a large current generator. The frequency is 50 Hz. The variation range is 50–1000 A, and the step is ~50 A. The experimental results are listed in Table 8. 

According to the data in Table 8, the average sensitivity of the discrete RC is 0.3634 mV/A and the average phase difference between the output voltage and the input current is 90.04°. 

According to the physical size of the discrete RC, the mutual inductance calculated using the MVP method is 1.0592 μH and the mutual inductance calculated using the finite element method is 1.0496 μH. The experimental value is 1.1567 μH.

The relationship between the sensitivity and the mutual inductance of the discrete RC can be expressed as follows:

(16) S=ωM=2πfM,

where *S* is the sensitivity of the discrete RC, *ω* is the angular frequency of the excitation source, *M* is the mutual inductance, and *f* is the power frequency. 

Table 9 lists the mutual inductance and sensitivity of the discrete RC obtained by using the MVP method, the finite element method, and the performance test, respectively. 

The results obtained from using the MVP method and the finite element method have some error compared with the experimental value, being, respectively, 8.42% and 9.27%. The main reasons are as follows. For both the MVP method and the finite element method, the idealized model of the discrete RC can only reflect the actual size of the coil but not fully reflect the actual complex structures; the real discrete RC has a certain discrepancy from the design scheme owing to the limitation of the production process. Moreover, influence factors in the test may affect the accuracy of measurement. However, the error between the actual measurement and the theoretical calculations does not affect the guiding role of the MVP method and the finite element method in the design and optimization of a discrete RC. 

The current signal collected by the discrete RC is actually the differential signal of the measured current with respect to time, so the actual output voltage leads the primary current by 90°. The average phase difference of the discrete RC is 90.04°. The absolute error is 0.04°. Factors causing the error in phase difference include the measurement system and the stray capacitance and inductance of the discrete RC. 

**Figure 20:**
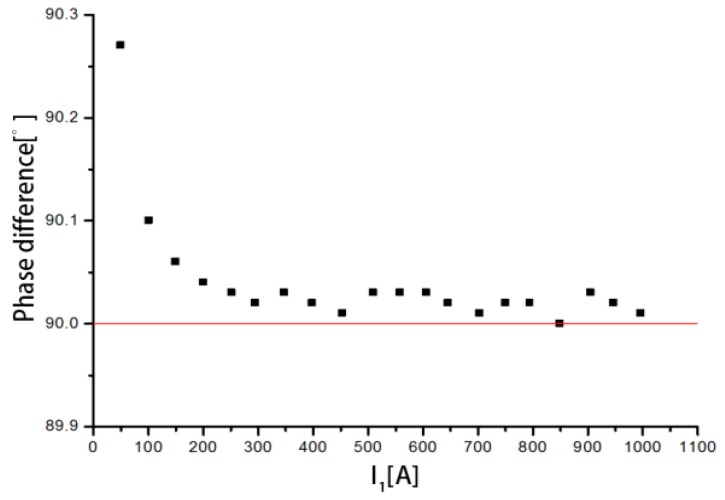
Distribution of the phase difference.

The phase differences between the output voltage and the input current are shown in Figure 20 when the measured current varies from 50 to 1000 A. It can be seen that the phase differences are all above 90°. When the measured current is 50.3146 A, the phase difference is 90.272°, which is the highest. With increasing current, the phase difference gradually stabilizes between 90° and 90.03°. In summary, the discrete RC has a large phase error in the measurement of small current, and the measurement accuracy is low. As the primary current increases, the measurement accuracy increases.

**Figure 21:**
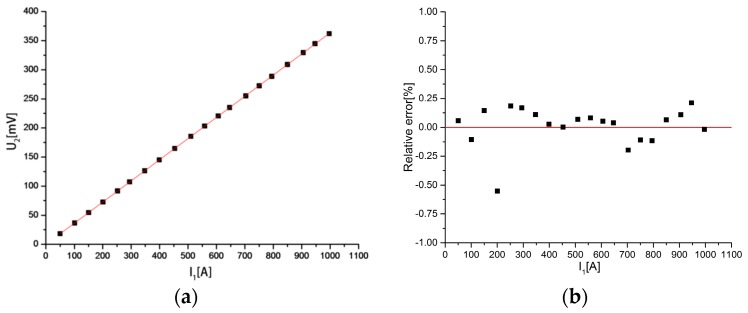
(**a**) Relationship between the output voltage; (**b**) Relative error between the measured current and the measured current and the primary current. Out consistency of the discrete RC.

Another performance indicator of the discrete RC is the output consistency. The linearity of output determines whether the discrete RC can truly reflect the waveform of the measured current. The relationship between the output voltage and the measured current is shown in Figure 21a. It can be seen that the relationship between the output voltage and the measured current is close to a straight line. Figure 21b is the relative error between the measured current and the primary current. It can be seen that the maximum relative error is only 0.55%, where *I*_1_ = 200.965 A.

#### 7.2.2. Rectangular Conductor Experiment

In this experiment, the cross-sectional size of the conductor is 15 × 3 mm^2^, and the primary current is generated by a large current generator. The frequency is 50 Hz. The variation range is 50–1000 A, and the step is approximately 50 A. The experimental results are listed in Table 10.

The average sensitivity of the coil is 0.3573 mV/A, and the average phase difference between the output voltage and the input current is 90.02°.

According to the physical size of the discrete RC, the mutual inductance calculated using the MVP method is 1.0510 μH and the mutual inductance calculated using the finite element method is 1.0410 μH. The experimental value is 1.1373 μH. Table 11 lists the mutual inductance and sensitivities obtained by using the MVP method, the finite element method, and the test circuit.

The results of the MVP method and the finite element method have some error compared with the experimental values, being, respectively, 7.58% and 8.48%. The correctness of the simulation results have been proved. The average phase difference of the RC is 90.02°. The absolute error is 0.02°.

**Figure 22:**
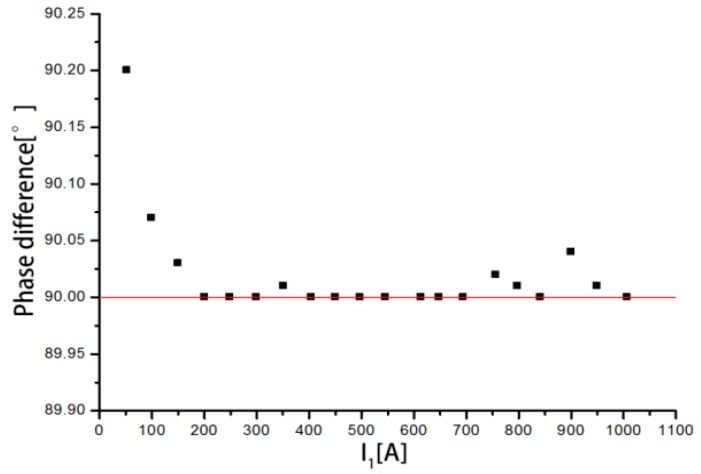
Distribution of the phase difference.

The phase differences between the output voltage and the input current are shown in Figure 22 when the measured current varies from 50 to 1000 A. It can be seen that the phase differences are all approximately 90°. When the measured current is 52.3401 A, the phase difference is 90.197°, which is the highest. With increasing current, the phase difference gradually stabilizes between 90° and 90.04°. In summary, the discrete RC has a large phase error in the measurement of small current, and the measurement accuracy is low. As the primary current increases, the measurement accuracy increases.

**Figure 23:**
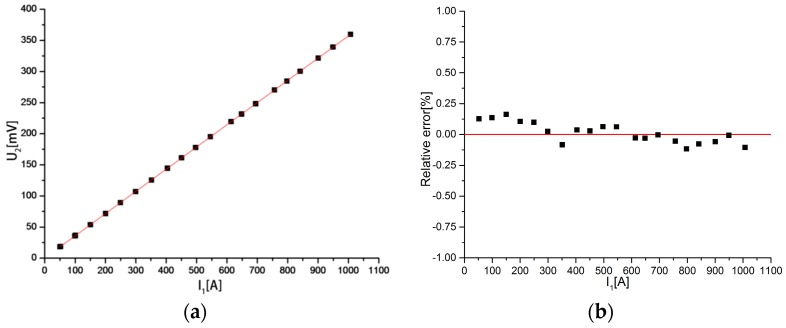
(**a**) Relationship between the output voltage; (**b**) Relative error between the measured current and the measured current and the primary current. Out consistency of the discrete RC.

The relationship between the output voltage and the measured current is shown in Figure 23a. It can be seen that the relationship between the output voltage and the measured current is close to a straight line. Figure 23b is the relative error between the measured current and the primary current. It can be seen that the maximum relative error is only 0.16%, where *I*_1_ = 150.121 A. 

### 7.3. Interference Test with an External Current Conductor

**Figure 24:**
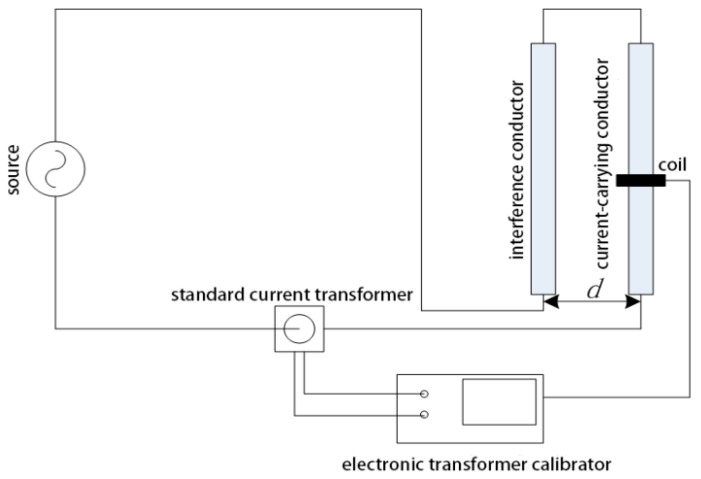
Test circuit.

To test the degree of interference from the external current conductor on the discrete RC, in this experiment, two identical rectangular conductors are placed in parallel: One is the conductor and the other is the external interference conductor. The test circuit is shown in Figure 24. 

The currents that flow through the conductor and the interference conductor are equal and opposite. *d* is the distance between the two parallel conductors. For *d* = 45 mm and *d* = 75 mm, the measurement results are listed in Table 12 and Table 13. 

According to the data in Table 12 and Table 13. The interference error decreases with the increase of the distance between current carrying conductor and external conductor. The experimental and simulation results are similar. 

## 8. Conclusions

In this paper, a new discrete RC with the advantages of flexible structure, low price and mass production was studied in detail. An MVP method was used to calculate the mutual inductance between the discrete RC and the conductor, and the correctness of this method has been verified by using the finite element method. 

The influence of section parameters, inclination, and eccentricity of the conductor on the accuracy of discrete RC has been calculated by MVP method, providing a reference for engineering applications. The influence of an external current on the interference error of the discrete RC is studied, and the results indicate that there are optimal values for the length, winding density, and position arrangements of the solenoid. The results also show that the eccentricity error and interference error of the discrete RC both decrease with increasing number of solenoids. 

A discrete RC prototype was devised and manufactured. The experimental results show consistent output characteristics, and the sensitivity and the mutual inductance calculated by using the MVP method and the finite element method are very close to the experimental results. The influence of an external current conductor on measurement of the discrete RC is analyzed via experiment. The results show that the degree of interference from an external current decreases with increasing distance between the external conductor and the measured conductor, which are consistent with the simulation results. 

## Figures and Tables

**Table 1:** Mutual inductance of the circular conductor calculated by using the MVP method.

Mutual Inductance	Rog1	Rog2	Rog3	Rog4	Rog
*M* [×10^−7^ H]	0.9543	2.3142	0.9543	2.3143	6.5370

**Table 2:** Mutual inductance of the circular conductor calculated by using the finite element method.

Mutual Inductance	Rog1	Rog2	Rog3	Rog4	Rog
*M* [×10^−7^ H]	0.9433	2.2863	0.9427	2.2880	6.4603

**Table 3:** Mutual inductance of the rectangular conductor calculated by using the MVP method.

Mutual Inductance	Rog1	Rog2	Rog3	Rog4	Rog
*M* [×10^−7^ H]	1.0612	2.1715	1.0612	2.1715	6.4655

**Table 4:** Mutual inductance of the rectangular conductor calculated by using the finite element method.

Mutual Inductance	Rog1	Rog2	Rog3	Rog4	Rog
*M* [×10^−7^ H]	1.0579	2.1355	1.0538	2.1398	6.3867

**Table 5:** Maximum interference errors (*L*_1_ = 6–20 mm).

*L*_1_ [mm]	6	8	10	12	14	16	18	20
Maximum interference error [%]	19.1	13.9	9.3	8.5	10.0	11.0	15.4	16.2

**Table 6:** Maximum interference errors (*ρ*_1_ = 10–80 turns/mm).

*ρ*_1_ [turns/mm]	10	20	30	40	50	60	70	80
Maximum interference error [%]	32.7	25.4	19.2	13.9	9.3	12.8	17.7	22.1

**Table 7:** Maximum interference errors (*d*_1_ = 8.5–12 mm).

*d*_1_ [mm]	8.5	9	9.5	10	10.5	11	11.5	12
Maximum interference error [%]	10.3	10.0	10.0	9.3	10.3	10.8	14.0	14.1

**Table 8:** Experimental results using the discrete RC for measuring the circular conductor.

Serial Number	Primary Current (RMS) *I*_1_ [A]	Secondary Voltage (RMS) *U*_2_ [mV]	Sensitivity *S* [mV·A^−1^]	Phase Difference [°]
1	50.315	18.295	0.3636	90.27
2	101.018	36.671	0.3630	90.10
3	150.225	54.671	0.3639	90.06
4	200.965	72.628	0.3614	90.04
5	251.859	91.694	0.3641	90.03
6	294.777	107.302	0.3640	90.02
7	347.395	126.382	0.3638	90.03
8	399.021	145.043	0.3635	90.02
9	453.020	164.631	0.3634	90.01
10	509.943	185.440	0.3636	90.03
11	558.572	203.151	0.3637	90.03
12	606.185	220.404	0.3636	90.03
13	646.508	235.030	0.3635	90.02
14	703.246	255.056	0.3627	90.01
15	750.460	272.420	0.3630	90.02
16	794.924	288.540	0.3630	90.02
17	849.889	309.047	0.3636	90.01
18	905.480	329.408	0.3638	90.01
19	946.824	344.807	0.3642	90.02
20	996.523	362.075	0.3633	90.01

**Table 9:** Results from the MVP method, the finite element method, and the experiment.

	MVP Method	Finite Element Method	Experimental Result
Mutual inductance *M* [μH]	1.0592	1.0496	1.1567
Sensitivity *S* [mV·A^−1^]	0.3328	0.3297	0.3634

**Table 10:** Experimental results using the discrete RC for measuring the rectangular conductor.

Serial Number	Primary Current (RMS) *I*_1_ [A]	Secondary Voltage (RMS) *U*_2_ [mV]	Sensitivity *S* [mV·A^−1^]	Phase Difference [°]
1	52.340	18.725	0.3577	90.20
2	99.137	35.470	0.3578	90.07
3	150.121	53.726	0.3579	90.03
4	200.232	71.619	0.3577	90.01
5	249.176	89.119	0.3577	90.00
6	299.091	106.892	0.3574	90.00
7	351.436	125.463	0.3570	90.01
8	403.993	144.400	0.3574	89.99
9	450.590	161.040	0.3574	90.00
10	496.993	177.687	0.3575	90.00
11	545.776	195.127	0.3575	90.00
12	613.740	219.226	0.3572	90.00
13	648.250	231.547	0.3572	90.00
14	694.619	248.179	0.3573	90.02
15	756.817	270.262	0.3571	90.01
16	797.323	284.550	0.3569	89.99
17	841.110	300.296	0.3570	90.03
18	900.568	321.584	0.3571	90.01
19	949.103	339.091	0.3573	90.00
20	1007.370	359.554	0.3569	90.00

**Table 11:** Results from the MVP method, the finite element method, and the experiment.

	MVP Method	Finite Element Method	Experimental Value
Mutual inductance *M* [μH]	1.0510	1.0410	1.1373
Sensitivity *S* [mV·A^−1^]	0.3302	0.3270	0.3573

**Table 12:** Experimental results (*d* = 45 mm).

Serial Number	Primary Current (RMS) *I*_1_ [A]	Secondary Voltage (RMS) *U*_2_ [mV]	Interference Error [%]
1	98.579	35.573	1.0
2	202.633	73.074	0.9
3	293.964	107.186	1.0
4	394.972	142.250	0.8
5	506.316	182.450	0.8
6	601.667	216.804	0.8
7	702.007	252.957	0.8
8	798.385	287.733	0.9
9	902.281	325.729	1.0
10	1000.160	360.959	1.0

**Table 13:** Experimental results (*d* = 75 mm).

Serial Number	Primary Current (RMS) *I*_1_ [A]	Secondary Voltage (RMS) *U*_2_ [mV]	Interference Error [%]
1	99.189	35.639	0.6
2	193.137	69.365	0.5
3	299.396	107.593	0.6
4	397.752	142.981	0.6
5	498.513	179.303	0.7
6	603.003	217.001	0.7
7	703.761	253.194	0.7
8	799.491	287.661	0.7
9	899.589	323.168	0.5
10	993.415	356.939	0.6

## References

[1] Ziegler S., Woodward R.C., Lu H.H.C., Borle L.J. (2009). Current Sensing Techniques: A Review. IEEE Sens. J..

[2] Rogowski W., Steinhaus W. (1912). Die messung der magnetischen spannung. Arch. Elektrotech..

[3] Marracci M., Tellini B., Zappacosta C., Robles G. (2011). Critical Parameters for Mutual Inductance between Rogowski Coil and Primary Conductor. IEEE Trans. Instrum. Meas..

[4] Dubickas V., Edin H. (2007). High-frequency model of the Rogowski coil with a small number of turns. IEEE Trans. Instrum. Meas..

[5] Chiampi M., Crotti G., Morando A. (2011). Evaluation of Flexible Rogowski Coil Performances in Power Frequency Applications. IEEE Trans. Instrum. Meas..

[6] Abdi-Jalebi E., Mc Mahon R. (2007). High-performance low-cost Rogowski transducers and accompanying circuitry. IEEE Trans. Instrum. Meas..

[7] Ferkovic L., Ilic D., Malaric R. (2009). Mutual Inductance of a Precise Rogowski Coil in Dependence of the Position of Primary Conductor. IEEE Trans. Instrum. Meas..

[8] Chen Q., Li H.-B., Zhang M.-M., Liu Y.-B. (2006). Design and characteristics of two Rogowski coils based on printed circuit board. IEEE Trans. Instrum. Meas..

[9] Budillon G., Buffat S., Houbre P., Toti-Buttin F. (2006). Electric Current Measuring Device, Current Sensor, Electric Trip Unit and Breaking Device Comprising Such a Measuring Device. U.S. Patent.

[10] Sun C., Yan J., Geng Y., Zhang K., Xu M., Liu J. Research on a novel current transducer based on discrete Rogowski coils. Proceedings of the International Conference on Condition Monitoring and Diagnosis.

[11] Rezaee M., Heydari H. Mutual inductance comparison in Rogowski coil with circular and rectangular cross-sections and its improvement. Proceedings of the 3rd IEEE Conference on Industrial Electronics and Applications.

[12] Murgatroyd P.N., Woodland D.N. Geometrical properties of Rogowski sensors. Proceedings of the IEE Colloquium on Low Frequency Power Measurement and Analysis.

[13] Strütt M. (1927). Das magnetische Feld eines rechteckigen, von Gleichstrom Leiters. Arch. Elektrotech..

[14] Ansys Inc. Maxwell 2D/3D Field Simulator v. 14. http://www.ansoft.com.

[15] Amoiralis E.I., Tsili M.A., Kladas A.G. (2009). Transformer Design and optimization: A literature survey. IEEE Trans. Power Deliv..

[16] Porto D., Bermudez J.L., Rachidi F. Design of a new air-cored current transformer: Analytical modeling and experimental validation. Proceedings of the 2004 IEEE Industry Applications Conference, 39th IAS Annual Meeting.

